# 4-Amino-3,5-bis­(2-pyrid­yl)-4*H*-1,2,4-triazole–benzene-1,2,3-tricarboxylic acid–water (1/1/2)

**DOI:** 10.1107/S1600536810010470

**Published:** 2010-04-14

**Authors:** Xiu-Juan Jiang

**Affiliations:** a School of Biological and Chemical Engineering, Jiaxing University, Zhejiang Jiaxing 314001, People’s Republic of China

## Abstract

Cocrystallization of 4-amino-3,5-bis­(2-pyrid­yl)-1,2,4-triazole (2-bpt) with hemimellitic acid (benzene-1,2,3-tricarboxylic acid) dihydrate (H_3_HMA·2H_2_O) produces the supra­molecular title compound, C_12_H_10_N_6_·C_9_H_6_O_6_·2H_2_O. Inter­molecular N—H⋯N hydrogen bonds are observed between the terminal pyridyl and amino groups of the 2-bpt molecule and the dihedral angles between the central ring and the pendant pyridine rings are 3.4 (7) and 13.8 (7)°. In the structure, homosynthons of graph set *R*_2_^2^(8) are observed to form centrosymmetric H_3_HMA dimers, which are extended into a two-dimensional supra­molecular layer *via* inter­molecular O—H⋯N and C—H⋯O hydrogen bonds and π–π stacking inter­actions [centroid–centroid distance = 3.541 (3) Å]. In addition, inter­layer uncoordinated water mol­ecules connect the layers through O—H⋯O hydrogen bonds, generating a three-dimensional network.

## Related literature

For background to the use of carboxylic acid in synthesis, see: Kuduva *et al.* (1999 ▶); Das *et al.* (2006 ▶). For the structure of trimesic acid, see: Biradha *et al.* (1998 ▶); Paz *et al.* (2003 ▶). For co-crystals of H_3_HMA, see: Dale *et al.* (2004 ▶); Du *et al.* (2005 ▶); For organic crystals of 4-amino-3,5-bis­(2-pyrid­yl)-1,2,4-triazole (2-bpt), see: Mernari *et al.* (1998 ▶); Ramos Silva *et al.* (2008 ▶). For the preparation of 2-bpt, see: Bentiss *et al.* (1999 ▶). 
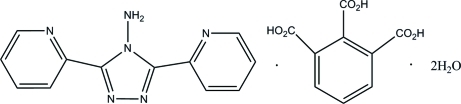


## Experimental

### 

#### Crystal data

C_12_H_10_N_6_·C_9_H_6_O_6_·2H_2_O
*M* *_r_* = 484.43
Triclinic, 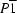

*a* = 8.4266 (10) Å
*b* = 8.6317 (10) Å
*c* = 15.7318 (18) Å
α = 75.152 (12)°
β = 77.179 (12)°
γ = 88.417 (13)°
*V* = 1078.0 (2) Å^3^
*Z* = 2
Mo *K*α radiation
μ = 0.12 mm^−1^
*T* = 294 K
0.24 × 0.21 × 0.18 mm

#### Data collection

Bruker APEXII CCD area-detector diffractometer
Absorption correction: multi-scan (*SADABS*; Bruker, 2001 ▶) *T*_min_ = 0.967, *T*_max_ = 0.980
5932 measured reflections
3765 independent reflections
2832 reflections with *I* > 2σ(*I*)
*R*_int_ = 0.018

#### Refinement

*R*[*F* ^2^ > 2σ(*F* ^2^)] = 0.037
*wR*(*F* ^2^) = 0.105
*S* = 1.05
3765 reflections
320 parameters
H-atom parameters constrained
Δρ_max_ = 0.18 e Å^−3^
Δρ_min_ = −0.16 e Å^−3^

### 

Data collection: *APEX2* (Bruker, 2003 ▶); cell refinement: *SAINT* (Bruker, 2001 ▶); data reduction: *SAINT*; program(s) used to solve structure: *SHELXS97* (Sheldrick, 2008 ▶); program(s) used to refine structure: *SHELXL97* (Sheldrick, 2008 ▶); molecular graphics: *SHELXTL* (Sheldrick, 2008 ▶) and *DIAMOND* (Brandenburg, 2005 ▶); software used to prepare material for publication: *SHELXTL*.

## Supplementary Material

Crystal structure: contains datablocks I, global. DOI: 10.1107/S1600536810010470/rz2426sup1.cif

Structure factors: contains datablocks I. DOI: 10.1107/S1600536810010470/rz2426Isup2.hkl

Additional supplementary materials:  crystallographic information; 3D view; checkCIF report

## Figures and Tables

**Table 1:** Hydrogen-bond geometry (Å, °)

*D*—H⋯*A*	*D*—H	H⋯*A*	*D*⋯*A*	*D*—H⋯*A*
O1—H1⋯O7^i^	0.82	1.79	2.600 (2)	171
O3—H3⋯N3^ii^	0.82	1.90	2.698 (2)	166
O5—H5⋯O6^iii^	0.82	1.85	2.674 (2)	177
N5—H5*A*⋯N6	0.90	2.08	2.786 (2)	134
N5—H5*B*⋯N1	0.90	2.17	2.804 (2)	127
O7—H7*A*⋯O8^iv^	0.85	1.92	2.766 (2)	172
O7—H7*B*⋯O4^v^	0.85	2.06	2.908 (2)	173
O8—H8*A*⋯N2	0.85	2.03	2.881 (2)	177
O8—H8*B*⋯O2^vi^	0.85	2.12	2.867 (2)	147
C14—H14⋯O8	0.93	2.51	3.348 (2)	149
C19—H19⋯O4^vii^	0.93	2.58	3.427 (2)	152
C20—H20⋯O6^viii^	0.93	2.47	3.386 (3)	167

Symmetry codes: (i) 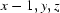; (ii) 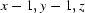; (iii) 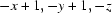; (iv) 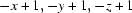; (v) 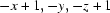; (vi) 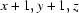; (vii) 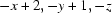; (viii) 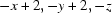.

**Fig. 1.**
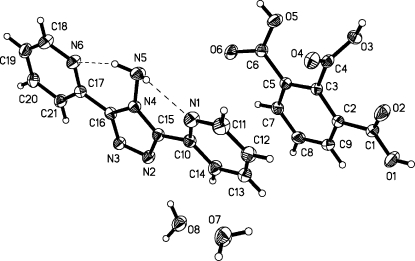
View of the molecular structure of the title compound, with displacement ellipsoids drawn at the 30% probability level (the dotted lines indicate intramolecular hydrogen bonds).

**Fig. 2.**
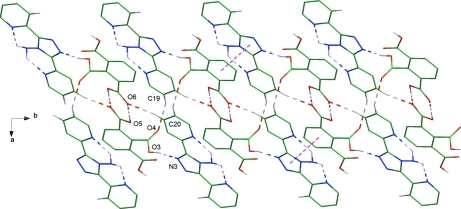
Perspective view of the supramolecular array of the title compound extending along the [010] direction (intrachain π–π interactions and hydrogen bonds are shown as dashed lines).

**Fig. 3.**
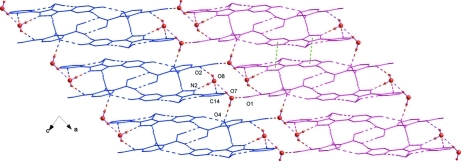
View of the 3-D supramolecular structure of the title compound (interchain π–π interactions and hydrogen bonds are shown as dashed lines).

## supplementary crystallographic information

### Comment

Carboxylic acid is one of the most commonly used functional groups in designing specific organic solids (Kuduva *et al.*, 1999; Das *et al.*, 2006). Compared with the well studied trimesic acid (benzene-1,3,5-tricarboxylic acid, H_3_TMA; Biradha *et al.*, 1998; Paz *et al.*, 2003), its isomer hemimellitic acid (benzene-1,2,3-tricarboxylic acid, H_3_HMA) has received little attention, with only few co-crystal structures reported to date (Dale *et al.*, 2004; Du *et al.*, 2005). As regards the angular dipyridyl derivative 4-amino-3,5-bis(2-pyridyl)-1,2,4-triazole (2-bpt), it can possibly provide multiple supramolecular interaction sites for molecular recognition, but organic crystals in relation to this component has rarely been studied up to now (Mernari *et al.*, 1998; Ramos Silva *et al.*, 2008). Herein the crystal structure of the title crystalline solid, 2-bpt.H_3_HMA.2H_2_O, is reported, which displays a 3-D supramolecular architecture and represents the new organic crystal for 2-bpt molecule.

The asymmetric unit of the title compound (Fig. 1) contains one 2-bpt, one H_3_HMA acid and two lattice water molecules. The dihedral angle between the 2-bpt and H_3_HMA rings is 5.4 (4)°. The two pyridyl groups of 2-bpt deviate by 10.7 (7)° from coplanarity and form dihedral angels of 3.4 (7) and 13.8 (7)°, respectively, with the central triazolyl ring. In the H_3_HMA molecule, the O3/C4/O4 carboxyl group is nearly perpendicular to the benzene plane (dihedral angle 81.4 (7)°), while the corresponding angles for the O1/C1/O2 and O5/C6/O6 groups are 7.8 (1) and 22.1 (8)°, respectively. In the 2-bpt molecule, N5—H5A···N6 and N5—H5B···N1 intramolecular hydrogen interactions are observed, as expected, between the terminal pyridyl and amino groups (Table 1). The O5—C6—O6 carboxyl groups of centrosymmetrically related H_3_HMA molecules form strong intermolecular hydrogen bonds, affording a dimeric unit with homosynthon of graph set *R*_2_^2^(8) (Table 1). Furthermore, the dimers connect adjacent 2-bpt molecules *via* the nearly perpendicular carboxyl groups (Table 1), generating a 1-D supramolecular array along the [010] direction (Fig. 2). In addition, intrachain C20—H20···O6 contacts (Table 1) and π–π stacking interactions (centroid-centroid distance = 3.541 (3) Å) extend the chains into a 2-D layer. The lattice water molecules occupy the interspaces of adjacent layers. The molecule including the O8 oxygen atom forms interlayer O8—H8A···N2, O8—H8B···O2 and C14—H14···O8 interactions, within which a *R*_2_^2^(7) synthon can be observed (Table 1); the water molecule including the O7 oxygen atom hydrogen-bonds adjacent layers and water molecules to finally afford a 3-D supramolecular structure (Table 1, Fig. 3). Examination of the interlayer solvent volume by *PLATON* (Spek, 2009) reveals a value of 81.0 Å^3^ (7.5% of the unit cell volume).

### Experimental

A mixture of 2-bpt (Bentiss *et al.*, 1999) (23.8 mg, 0.1 mmol), H_3_HMA.2H_2_O (24.6 mg, 0.1 mmol) and water (10 ml) was sealed in a Teflon-lined stainless steel vessel (20 ml), which was heated at 413 K for three days and then cooled to room temperature. Colourless block single crystals of the title compound were obtained in 52% yield (25.0 mg, based on 2-bpt). Anal. Calcd for C_21_H_20_N_6_O_8_: C, 52.07; H, 4.16, N, 17.35. Found: C, 52.16; H, 4.08, N, 17.25%. IR (cm^-1^): 3504*s*, 3275*s*, 1715*vs*, 1687*vs*, 1586*s*, 1559*s*, 1466*s*, 1412*m*, 1254*vs*, 1154*s*, 1076*m*, 1001*s*, 957*m*, 893*m*, 794*s*, 741*s*, 699*m*, 674*s*, 585*m*, 545*m*.

### Refinement

The water and amine H atoms were located in a difference Fourier map and refined with the O—H and N—H bond lengths constrained to 0.85 and 0.90 Å, respectively, and with *U*_iso_(H) = 1.5*U*_eq_(O) and 1.2*U*_eq_(N). All other H atoms were placed at calculated positions and refined as riding, with C—H = 0.93 Å, O—H = 0.82 Å, and with *U*_iso_(H) = 1.2*U*_eq_(C) or 1.5*U*_eq_(O).

### Crystal data

**Table:**

C_12_H_10_N_6_·C_9_H_6_O_6_·2H_2_O	*Z* = 2
*M**_r_* = 484.43	*F*(000) = 504
Triclinic, *P*1	*D*_x_ = 1.492 Mg m^−^^3^
Hall symbol: -P 1	Mo *K*α radiation, λ = 0.71073 Å
*a* = 8.4266 (10) Å	Cell parameters from 2173 reflections
*b* = 8.6317 (10) Å	θ = 2.8–25.9°
*c* = 15.7318 (18) Å	µ = 0.12 mm^−^^1^
α = 75.152 (12)°	*T* = 294 K
β = 77.179 (12)°	Block, colourless
γ = 88.417 (13)°	0.24 × 0.21 × 0.18 mm
*V* = 1078.0 (2) Å^3^	

### Data collection

**Table:**

Bruker APEXII CCD area-detector diffractometer	3765 independent reflections
Radiation source: fine-focus sealed tube	2832 reflections with *I* > 2σ(*I*)
graphite	*R*_int_ = 0.018
phi and ω scans	θ_max_ = 25.0°, θ_min_ = 2.4°
Absorption correction: multi-scan (*SADABS*; Bruker, 2001)	*h* = −10→9
*T*_min_ = 0.967, *T*_max_ = 0.980	*k* = −9→10
5932 measured reflections	*l* = −18→18

### Refinement

**Table:**

Refinement on *F*^2^	Secondary atom site location: difference Fourier map
Least-squares matrix: full	Hydrogen site location: inferred from neighbouring sites
*R*[*F*^2^ > 2σ(*F*^2^)] = 0.037	H-atom parameters constrained
*wR*(*F*^2^) = 0.105	*w* = 1/[σ^2^(*F*_o_^2^) + (0.0552*P*)^2^ + 0.1197*P*] where *P* = (*F*_o_^2^ + 2*F*_c_^2^)/3
*S* = 1.05	(Δ/σ)_max_ < 0.001
3765 reflections	Δρ_max_ = 0.18 e Å^−^^3^
320 parameters	Δρ_min_ = −0.16 e Å^−^^3^
0 restraints	Extinction correction: *SHELXL97* (Sheldrick, 2008), Fc^*^=kFc[1+0.001xFc^2^λ^3^/sin(2θ)]^-1/4^
Primary atom site location: structure-invariant direct methods	Extinction coefficient: 0.021 (2)

### Special details

**Table:**

Geometry. All esds (except the esd in the dihedral angle between two l.s. planes) are estimated using the full covariance matrix. The cell esds are taken into account individually in the estimation of esds in distances, angles and torsion angles; correlations between esds in cell parameters are only used when they are defined by crystal symmetry. An approximate (isotropic) treatment of cell esds is used for estimating esds involving l.s. planes.
Refinement. Refinement of *F*^2^ against ALL reflections. The weighted *R*-factor wR and goodness of fit *S* are based on *F*^2^, conventional *R*-factors *R* are based on *F*, with *F* set to zero for negative *F*^2^. The threshold expression of *F*^2^ > σ(*F*^2^) is used only for calculating *R*-factors(gt) etc. and is not relevant to the choice of reflections for refinement. *R*-factors based on *F*^2^ are statistically about twice as large as those based on *F*, and *R*- factors based on ALL data will be even larger.

### Fractional atomic coordinates and isotropic or equivalent isotropic displacement parameters (Å^2^)

**Table:**

	*x*	*y*	*z*	*U*_iso_*/*U*_eq_	
O1	−0.15210 (17)	0.29442 (17)	0.47310 (9)	0.0579 (4)	
H1	−0.2077	0.2167	0.5064	0.087*	
O2	−0.05082 (17)	0.11617 (16)	0.39702 (9)	0.0569 (4)	
O3	0.02722 (15)	0.19395 (14)	0.20039 (9)	0.0415 (3)	
H3	0.0232	0.1036	0.1930	0.062*	
O4	0.24886 (16)	0.10468 (15)	0.25232 (9)	0.0477 (3)	
O5	0.32592 (17)	0.38341 (17)	0.07961 (9)	0.0548 (4)	
H5	0.3873	0.3949	0.0299	0.082*	
O6	0.48197 (16)	0.58328 (16)	0.08438 (9)	0.0546 (4)	
N1	0.7015 (2)	0.42032 (18)	0.25775 (11)	0.0484 (4)	
N2	0.83363 (18)	0.82310 (17)	0.24200 (10)	0.0402 (4)	
N3	0.95863 (17)	0.90648 (17)	0.17665 (10)	0.0387 (4)	
N4	0.91424 (17)	0.68331 (16)	0.14315 (9)	0.0355 (3)	
N5	0.9121 (2)	0.56814 (18)	0.09338 (10)	0.0482 (4)	
H5A	1.0131	0.5743	0.0583	0.058*	
H5B	0.8885	0.4727	0.1340	0.058*	
N6	1.18042 (19)	0.76103 (18)	−0.01199 (10)	0.0462 (4)	
C1	−0.0538 (2)	0.2505 (2)	0.40647 (12)	0.0395 (4)	
C2	0.0538 (2)	0.3858 (2)	0.34279 (11)	0.0353 (4)	
C3	0.1524 (2)	0.3644 (2)	0.26222 (11)	0.0332 (4)	
C4	0.1486 (2)	0.2056 (2)	0.23849 (11)	0.0353 (4)	
C5	0.2564 (2)	0.4922 (2)	0.20656 (11)	0.0341 (4)	
C6	0.3642 (2)	0.4873 (2)	0.11777 (12)	0.0385 (4)	
C7	0.2594 (2)	0.6357 (2)	0.23120 (12)	0.0410 (4)	
H7	0.3308	0.7187	0.1945	0.049*	
C8	0.1584 (2)	0.6570 (2)	0.30912 (12)	0.0435 (5)	
H8	0.1589	0.7545	0.3240	0.052*	
C9	0.0569 (2)	0.5320 (2)	0.36447 (12)	0.0415 (4)	
H9	−0.0108	0.5455	0.4173	0.050*	
C10	0.6861 (2)	0.5630 (2)	0.27668 (11)	0.0370 (4)	
C11	0.5973 (3)	0.3031 (2)	0.31086 (14)	0.0565 (6)	
H11	0.6059	0.2032	0.2984	0.068*	
C12	0.4780 (3)	0.3209 (2)	0.38293 (13)	0.0529 (5)	
H12	0.4092	0.2351	0.4185	0.063*	
C13	0.4633 (2)	0.4678 (3)	0.40076 (13)	0.0512 (5)	
H13	0.3834	0.4843	0.4486	0.061*	
C14	0.5686 (2)	0.5914 (2)	0.34694 (13)	0.0494 (5)	
H14	0.5606	0.6926	0.3578	0.059*	
C15	0.8071 (2)	0.6891 (2)	0.22104 (11)	0.0353 (4)	
C16	1.0074 (2)	0.8208 (2)	0.11698 (11)	0.0347 (4)	
C17	1.1425 (2)	0.8672 (2)	0.03777 (11)	0.0350 (4)	
C18	1.3039 (2)	0.8006 (3)	−0.08423 (13)	0.0528 (5)	
H18	1.3313	0.7281	−0.1194	0.063*	
C19	1.3924 (2)	0.9422 (3)	−0.10926 (14)	0.0516 (5)	
H19	1.4777	0.9646	−0.1600	0.062*	
C20	1.3524 (2)	1.0498 (3)	−0.05799 (13)	0.0499 (5)	
H20	1.4103	1.1469	−0.0736	0.060*	
C21	1.2256 (2)	1.0132 (2)	0.01692 (13)	0.0454 (5)	
H21	1.1965	1.0847	0.0526	0.054*	
O7	0.65104 (17)	0.06876 (17)	0.58459 (10)	0.0643 (4)	
H7A	0.5494	0.0662	0.5868	0.096*	
H7B	0.6834	0.0121	0.6299	0.096*	
O8	0.68068 (17)	0.91230 (18)	0.40477 (10)	0.0669 (5)	
H8A	0.7248	0.8889	0.3557	0.100*	
H8B	0.7366	0.9709	0.4240	0.100*	

### Atomic displacement parameters (Å^2^)

**Table:**

	*U*^11^	*U*^22^	*U*^33^	*U*^12^	*U*^13^	*U*^23^
O1	0.0631 (9)	0.0537 (9)	0.0466 (8)	−0.0169 (7)	0.0181 (7)	−0.0186 (7)
O2	0.0668 (9)	0.0442 (8)	0.0499 (8)	−0.0155 (7)	0.0125 (7)	−0.0144 (7)
O3	0.0465 (7)	0.0343 (7)	0.0474 (7)	−0.0019 (6)	−0.0106 (6)	−0.0166 (6)
O4	0.0462 (8)	0.0411 (8)	0.0517 (8)	0.0069 (6)	−0.0058 (6)	−0.0096 (6)
O5	0.0656 (9)	0.0533 (9)	0.0392 (8)	−0.0188 (7)	0.0109 (6)	−0.0179 (7)
O6	0.0509 (8)	0.0587 (9)	0.0459 (8)	−0.0208 (7)	0.0083 (6)	−0.0128 (7)
N1	0.0552 (10)	0.0360 (9)	0.0493 (10)	−0.0050 (8)	−0.0032 (8)	−0.0091 (7)
N2	0.0413 (8)	0.0344 (8)	0.0413 (9)	−0.0055 (7)	0.0000 (7)	−0.0108 (7)
N3	0.0408 (8)	0.0327 (8)	0.0403 (8)	−0.0040 (7)	−0.0027 (7)	−0.0103 (7)
N4	0.0392 (8)	0.0311 (8)	0.0369 (8)	−0.0028 (6)	−0.0059 (6)	−0.0115 (6)
N5	0.0577 (10)	0.0433 (9)	0.0448 (9)	−0.0134 (8)	−0.0009 (8)	−0.0210 (7)
N6	0.0503 (9)	0.0411 (9)	0.0434 (9)	−0.0009 (7)	0.0023 (7)	−0.0147 (7)
C1	0.0402 (10)	0.0446 (11)	0.0321 (10)	−0.0070 (8)	−0.0016 (8)	−0.0114 (8)
C2	0.0355 (9)	0.0389 (10)	0.0318 (9)	−0.0044 (8)	−0.0067 (7)	−0.0096 (8)
C3	0.0327 (9)	0.0347 (10)	0.0316 (9)	−0.0026 (7)	−0.0065 (7)	−0.0074 (7)
C4	0.0368 (10)	0.0344 (10)	0.0300 (9)	−0.0030 (8)	0.0004 (8)	−0.0063 (7)
C5	0.0335 (9)	0.0365 (10)	0.0311 (9)	−0.0025 (8)	−0.0066 (7)	−0.0069 (7)
C6	0.0400 (10)	0.0364 (10)	0.0353 (10)	−0.0053 (8)	−0.0039 (8)	−0.0055 (8)
C7	0.0432 (10)	0.0394 (11)	0.0382 (10)	−0.0106 (8)	−0.0072 (8)	−0.0066 (8)
C8	0.0524 (11)	0.0391 (11)	0.0426 (11)	−0.0062 (9)	−0.0104 (9)	−0.0159 (9)
C9	0.0434 (10)	0.0495 (12)	0.0336 (10)	−0.0051 (9)	−0.0047 (8)	−0.0166 (8)
C10	0.0384 (10)	0.0349 (10)	0.0367 (10)	−0.0023 (8)	−0.0083 (8)	−0.0069 (8)
C11	0.0714 (15)	0.0356 (11)	0.0556 (13)	−0.0132 (10)	−0.0043 (11)	−0.0066 (9)
C12	0.0592 (13)	0.0483 (12)	0.0446 (12)	−0.0186 (10)	−0.0076 (10)	−0.0017 (9)
C13	0.0458 (11)	0.0630 (14)	0.0423 (11)	−0.0128 (10)	−0.0021 (9)	−0.0141 (10)
C14	0.0471 (11)	0.0468 (12)	0.0525 (12)	−0.0096 (9)	0.0001 (9)	−0.0178 (10)
C15	0.0350 (9)	0.0324 (10)	0.0375 (10)	−0.0007 (8)	−0.0062 (8)	−0.0087 (8)
C16	0.0368 (9)	0.0290 (9)	0.0380 (10)	−0.0018 (8)	−0.0081 (8)	−0.0081 (8)
C17	0.0354 (9)	0.0335 (10)	0.0351 (9)	0.0010 (8)	−0.0082 (7)	−0.0069 (8)
C18	0.0558 (12)	0.0521 (13)	0.0470 (12)	0.0038 (10)	0.0016 (10)	−0.0181 (10)
C19	0.0411 (11)	0.0606 (14)	0.0463 (12)	−0.0011 (10)	0.0005 (9)	−0.0100 (10)
C20	0.0399 (11)	0.0532 (12)	0.0516 (12)	−0.0117 (9)	−0.0026 (9)	−0.0099 (10)
C21	0.0430 (11)	0.0461 (11)	0.0466 (11)	−0.0055 (9)	−0.0032 (9)	−0.0160 (9)
O7	0.0588 (9)	0.0635 (10)	0.0571 (9)	−0.0175 (8)	−0.0048 (7)	0.0036 (7)
O8	0.0636 (10)	0.0714 (10)	0.0649 (10)	−0.0227 (8)	0.0080 (8)	−0.0329 (8)

### Geometric parameters (Å, °)

**Table:**

O1—C1	1.315 (2)	C7—C8	1.380 (2)
O1—H1	0.8200	C7—H7	0.9300
O2—C1	1.205 (2)	C8—C9	1.376 (2)
O3—C4	1.313 (2)	C8—H8	0.9300
O3—H3	0.8200	C9—H9	0.9300
O4—C4	1.210 (2)	C10—C14	1.379 (3)
O5—C6	1.283 (2)	C10—C15	1.472 (2)
O5—H5	0.8200	C11—C12	1.377 (3)
O6—C6	1.240 (2)	C11—H11	0.9300
N1—C11	1.335 (2)	C12—C13	1.364 (3)
N1—C10	1.336 (2)	C12—H12	0.9300
N2—C15	1.319 (2)	C13—C14	1.378 (3)
N2—N3	1.3661 (19)	C13—H13	0.9300
N3—C16	1.328 (2)	C14—H14	0.9300
N4—C16	1.361 (2)	C16—C17	1.466 (2)
N4—C15	1.363 (2)	C17—C21	1.386 (2)
N4—N5	1.4169 (19)	C18—C19	1.370 (3)
N5—H5A	0.9000	C18—H18	0.9300
N5—H5B	0.9000	C19—C20	1.369 (3)
N6—C18	1.337 (2)	C19—H19	0.9300
N6—C17	1.340 (2)	C20—C21	1.378 (3)
C1—C2	1.498 (2)	C20—H20	0.9300
C2—C9	1.391 (2)	C21—H21	0.9300
C2—C3	1.406 (2)	O7—H7A	0.8500
C3—C5	1.404 (2)	O7—H7B	0.8499
C3—C4	1.513 (2)	O8—H8A	0.8510
C5—C7	1.392 (2)	O8—H8B	0.8502
C5—C6	1.498 (2)		
			
C1—O1—H1	109.5	C2—C9—H9	119.4
C4—O3—H3	109.5	N1—C10—C14	122.72 (16)
C6—O5—H5	109.5	N1—C10—C15	116.49 (15)
C11—N1—C10	116.81 (16)	C14—C10—C15	120.73 (16)
C15—N2—N3	107.75 (13)	N1—C11—C12	124.13 (19)
C16—N3—N2	108.01 (13)	N1—C11—H11	117.9
C16—N4—C15	106.25 (14)	C12—C11—H11	117.9
C16—N4—N5	127.04 (14)	C13—C12—C11	118.18 (18)
C15—N4—N5	126.14 (13)	C13—C12—H12	120.9
N4—N5—H5A	104.7	C11—C12—H12	120.9
N4—N5—H5B	106.5	C12—C13—C14	119.09 (19)
H5A—N5—H5B	112.9	C12—C13—H13	120.5
C18—N6—C17	117.34 (16)	C14—C13—H13	120.5
O2—C1—O1	123.81 (16)	C13—C14—C10	119.07 (18)
O2—C1—C2	123.40 (16)	C13—C14—H14	120.5
O1—C1—C2	112.79 (16)	C10—C14—H14	120.5
C9—C2—C3	120.26 (16)	N2—C15—N4	109.31 (14)
C9—C2—C1	119.61 (15)	N2—C15—C10	124.55 (15)
C3—C2—C1	120.12 (15)	N4—C15—C10	126.07 (15)
C5—C3—C2	118.13 (15)	N3—C16—N4	108.69 (14)
C5—C3—C4	121.57 (14)	N3—C16—C17	124.82 (15)
C2—C3—C4	120.28 (14)	N4—C16—C17	126.47 (15)
O4—C4—O3	125.30 (16)	N6—C17—C21	122.64 (16)
O4—C4—C3	122.76 (16)	N6—C17—C16	116.38 (15)
O3—C4—C3	111.93 (15)	C21—C17—C16	120.97 (16)
C7—C5—C3	120.19 (15)	N6—C18—C19	123.60 (18)
C7—C5—C6	116.12 (15)	N6—C18—H18	118.2
C3—C5—C6	123.66 (15)	C19—C18—H18	118.2
O6—C6—O5	123.63 (16)	C20—C19—C18	118.55 (18)
O6—C6—C5	119.51 (16)	C20—C19—H19	120.7
O5—C6—C5	116.85 (14)	C18—C19—H19	120.7
C8—C7—C5	121.14 (16)	C19—C20—C21	119.48 (18)
C8—C7—H7	119.4	C19—C20—H20	120.3
C5—C7—H7	119.4	C21—C20—H20	120.3
C9—C8—C7	119.08 (17)	C20—C21—C17	118.39 (18)
C9—C8—H8	120.5	C20—C21—H21	120.8
C7—C8—H8	120.5	C17—C21—H21	120.8
C8—C9—C2	121.15 (16)	H7A—O7—H7B	117.1
C8—C9—H9	119.4	H8A—O8—H8B	117.1
			
C15—N2—N3—C16	−0.51 (19)	C12—C13—C14—C10	−0.1 (3)
O2—C1—C2—C9	−172.13 (19)	N1—C10—C14—C13	0.7 (3)
O1—C1—C2—C9	8.4 (2)	C15—C10—C14—C13	−176.28 (18)
O2—C1—C2—C3	7.0 (3)	N3—N2—C15—N4	0.5 (2)
O1—C1—C2—C3	−172.47 (16)	N3—N2—C15—C10	177.44 (16)
C9—C2—C3—C5	1.9 (3)	C16—N4—C15—N2	−0.29 (19)
C1—C2—C3—C5	−177.24 (15)	N5—N4—C15—N2	−172.11 (16)
C9—C2—C3—C4	−179.82 (16)	C16—N4—C15—C10	−177.18 (17)
C1—C2—C3—C4	1.0 (2)	N5—N4—C15—C10	11.0 (3)
C5—C3—C4—O4	80.1 (2)	N1—C10—C15—N2	−164.14 (17)
C2—C3—C4—O4	−98.1 (2)	C14—C10—C15—N2	13.0 (3)
C5—C3—C4—O3	−98.72 (18)	N1—C10—C15—N4	12.3 (3)
C2—C3—C4—O3	83.09 (19)	C14—C10—C15—N4	−170.58 (18)
C2—C3—C5—C7	−0.4 (3)	N2—N3—C16—N4	0.33 (19)
C4—C3—C5—C7	−178.66 (17)	N2—N3—C16—C17	−178.23 (16)
C2—C3—C5—C6	−178.20 (16)	C15—N4—C16—N3	−0.03 (19)
C4—C3—C5—C6	3.6 (3)	N5—N4—C16—N3	171.69 (16)
C7—C5—C6—O6	21.5 (3)	C15—N4—C16—C17	178.50 (17)
C3—C5—C6—O6	−160.65 (17)	N5—N4—C16—C17	−9.8 (3)
C7—C5—C6—O5	−157.19 (17)	C18—N6—C17—C21	0.0 (3)
C3—C5—C6—O5	20.7 (3)	C18—N6—C17—C16	−179.59 (17)
C3—C5—C7—C8	−1.6 (3)	N3—C16—C17—N6	176.17 (17)
C6—C5—C7—C8	176.36 (16)	N4—C16—C17—N6	−2.1 (3)
C5—C7—C8—C9	2.0 (3)	N3—C16—C17—C21	−3.5 (3)
C7—C8—C9—C2	−0.5 (3)	N4—C16—C17—C21	178.23 (17)
C3—C2—C9—C8	−1.5 (3)	C17—N6—C18—C19	0.1 (3)
C1—C2—C9—C8	177.70 (17)	N6—C18—C19—C20	−0.2 (3)
C11—N1—C10—C14	−0.4 (3)	C18—C19—C20—C21	0.1 (3)
C11—N1—C10—C15	176.69 (17)	C19—C20—C21—C17	0.0 (3)
C10—N1—C11—C12	−0.4 (3)	N6—C17—C21—C20	−0.1 (3)
N1—C11—C12—C13	0.9 (3)	C16—C17—C21—C20	179.49 (17)
C11—C12—C13—C14	−0.6 (3)		

### Hydrogen-bond geometry (Å, °)

**Table:**

*D*—H···*A*	*D*—H	H···*A*	*D*···*A*	*D*—H···*A*
O1—H1···O7^i^	0.82	1.79	2.600 (2)	171
O3—H3···N3^ii^	0.82	1.90	2.698 (2)	166
O5—H5···O6^iii^	0.82	1.85	2.674 (2)	177
N5—H5A···N6	0.90	2.08	2.786 (2)	134
N5—H5B···N1	0.90	2.17	2.804 (2)	127
O7—H7A···O8^iv^	0.85	1.92	2.766 (2)	172
O7—H7B···O4^v^	0.85	2.06	2.908 (2)	173
O8—H8A···N2	0.85	2.03	2.881 (2)	177
O8—H8B···O2^vi^	0.85	2.12	2.867 (2)	147
C14—H14···O8	0.93	2.51	3.348 (2)	149
C19—H19···O4^vii^	0.93	2.58	3.427 (2)	152
C20—H20···O6^viii^	0.93	2.47	3.386 (3)	167

Symmetry codes: (i) *x*−1, *y*, *z*; (ii) *x*−1, *y*−1, *z*; (iii) −*x*+1, −*y*+1, −*z*; (iv) −*x*+1, −*y*+1, −*z*+1; (v) −*x*+1, −*y*, −*z*+1; (vi) *x*+1, *y*+1, *z*; (vii) −*x*+2, −*y*+1, −*z*; (viii) −*x*+2, −*y*+2, −*z*.
